# Sweet syndrome associated with secondary nodular syphilis in an immunocompetent patient^☆^^☆☆^

**DOI:** 10.1016/j.abd.2020.06.017

**Published:** 2021-03-16

**Authors:** Claudio Escanilla, Yerco Goldman, Francisco Bobadilla, Laura Segovia

**Affiliations:** a Dermatology Unit, Barros Luco Hospital, Santiago de Chile, Chile; b Department of Dermatology, Faculty of Medicine, University of Chile, Santiago de Chile, Chile; c Pathology Anatomy Unit, Barros Luco Hospital, Santiago de Chile, Chile

**Keywords:** Sexually transmitted diseases, Sweet syndrome, Syphilis, Young adult

## Abstract

Sweet syndrome is an inflammatory disease characterized by fever, neutrophilia, papules and erythematous plaques, and a skin neutrophilic infiltrate. Syphilis has been reported among the infectious causes of Sweet syndrome. Syphilis can present atypical manifestations; a rare presentation is nodular syphilis, characterized by nodules with granulomas and plasma cells at histopathology. This case report presents a 20-year-old woman patient, with plaques and nodules, and systemic symptoms. The histopathological exam revealed both non-tuberculoid granulomas and a dense infiltration of polymorphonuclear neutrophils in the dermis. These findings, plus laboratory abnormalities, characteristic of both conditions, were conclusive for Sweet syndrome and nodular syphilis association.

## Introduction

Sweet Syndrome is a rare inflammatory disease of rapid progression characterized by fever, neutrophilia, painful erythematous papules, or plaques accompanied by a dense neutrophilic infiltrate on the papillary dermis without vasculitis.^1^ In general, it is classified in three settings: classic, malignancy-associated, and drug-induced. Classical Sweet Syndrome (SS) integrates the idiopathic SS, associated with vaccination, inflammatory disorders, pregnancy, or infection.^2^ Among the infectious causes, syphilis has been hardly reported.^3^ Besides typical exanthema and lesions on the mucous membrane, secondary syphilis may present atypical manifestations which imitate various dermatoses.^4^ An unusual clinical presentation is Nodular syphilis, characterized by nodules and plaques whose histology shows granulomas and plasma cells, with nearly 30 reported cases.^5^ Another infrequent presentation is the appearance of rapidly evolving erythematous plaques similar to SS.^6^, ^7^, ^8^

This case is presented since there are few reports published in the literature on the SS and nodular syphilis association.

## Case report

**Figure 1:**
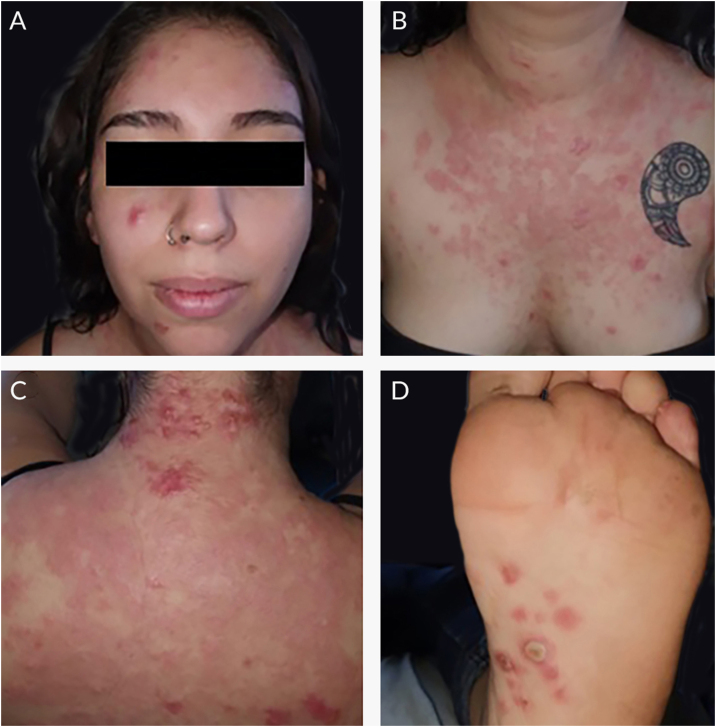
(A, B and C) Papules, plaques, and erythematous nodules on the face, chest, and back. (D) Erythematous papules and pustules on the foot sole.

**Figure 2:**
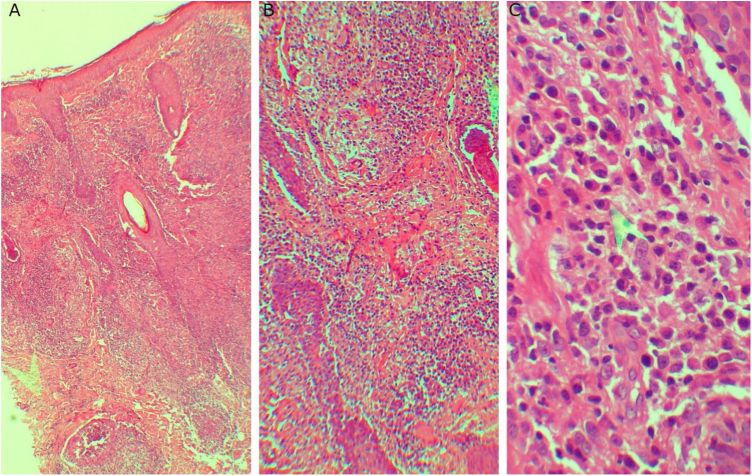
(A and B) Superficial and deep non-tuberculoid granulomatous dermatitis (A, Hematoxylin & eosin, ×40, B, Hematoxylin & eosin, ×100). (C) The pointer shows the presence of plasma cells (C, Hematoxylin & eosin, ×400).

**Figure 3:**
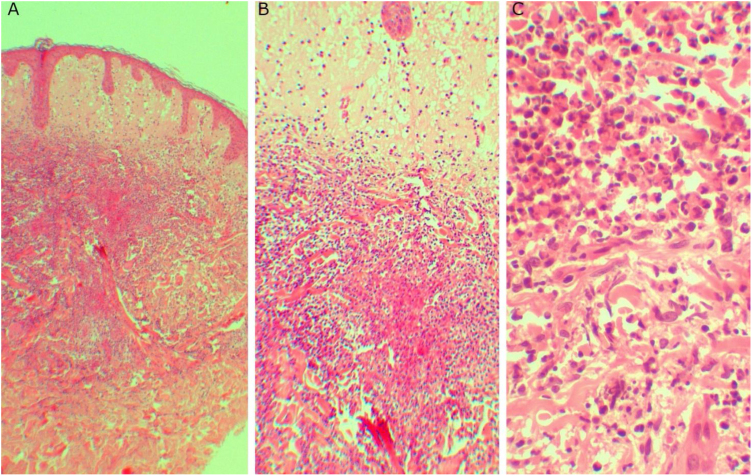
(A and B) Subepidermal edema associated with an inflammatory infiltrate on superficial and middle dermis (A, Hematoxylin & eosin, ×40, B, Hematoxylin & eosin, ×100). (C) Infiltrate composed of polymorphonuclear neutrophilis (C, Hematoxylin & eosin, ×400).

**Figure 4:**
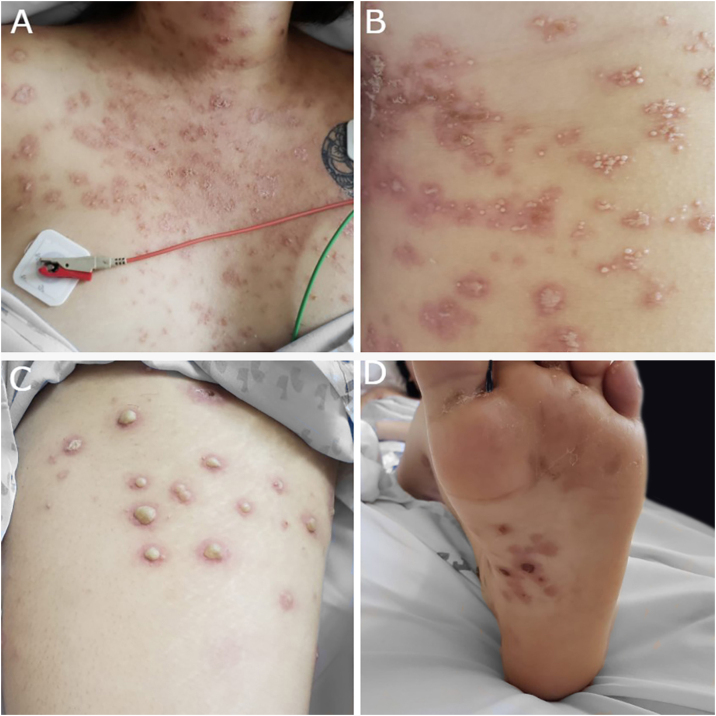
(A) Papules and plaques on chest with peeling and pustules. (B) Pustules on abdomen associated with erythema. (C) Huge pustules on thigh sur erythematous base. (D) Hemorrhagic crusts on the foot sole.

**Figure 5:**
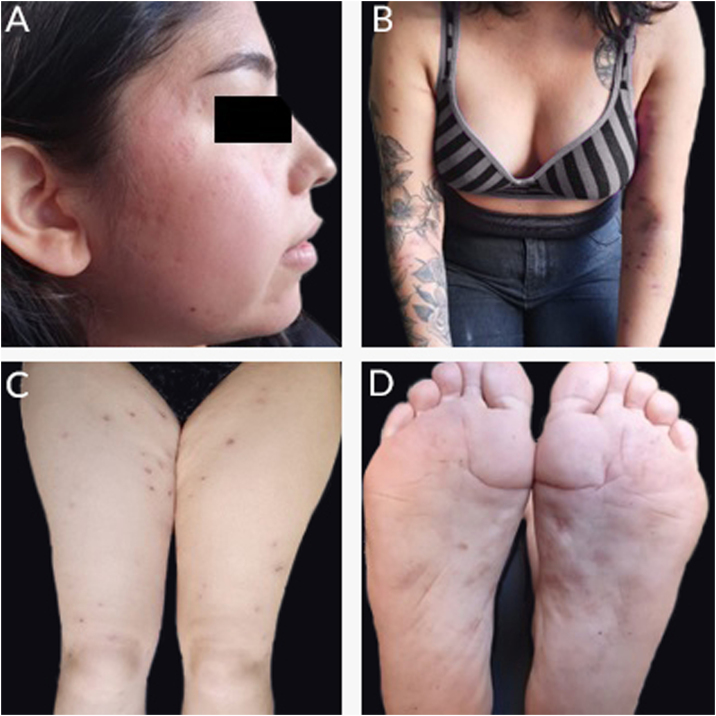
(A) Atrophic scars on the face. (B, C and D) Erythematous scars of anetodermic aspect on arms, thighs and feet.

A 20-year-old woman, without previous morbid history, presented for 20-days painful and progressive extension of cutaneous lesions, starting on the face and subsequent involvement of the trunk and extremities, related to noticeable general systemic symptoms, such as headache, and fever. On the physical examination, she presents numerous papules, plaques, and erythematous nodules on the face, chest and back, abdomen, forearm, muscles, ankles, palms of the hands and the soles of her feet, some vesicular and pustular lesions in the abdomen and ankle, presence of pink-erythematous lesion on the vaginal opening (Fig. 1). Given the suspicion of neutrophilic dermatosis, skin incisional biopsies were performed in the left temple region that showed superficial and deep non-tuberculoid granulomatous dermatitis, with the presence of plasma cells (Fig. 2), and in the abdominal region that showed noticeable subepidermal edema associated with dense infiltrate of polymorphonuclear neutrophils in the superficial and middle dermis, without vasculitis (Fig. 3). Hospitalization and blood tests were indicated: Hemoglobin: 11.3 g/dL, leukocytes: 17.300 µL with the segmentation of 86%, CRP: 329 mg/L, HIV: non-reactive, HbsAg: non-reactive, HCV: non-reactive. VDRL: reactive 1/16 dilution, MHA-TP: reactive. With these findings, she was diagnosed with secondary syphilis associated with the SS. Benzathine penicillin G 2.4 MUI twice a week and prednisone 1 mg/kg per day were prescribed. After the first dose of penicillin, the patient reacted with fever, tachycardia, and increased inflammatory parameters, which were interpreted as a Jarisch Herxheimer reaction, when most of the lesions took a pustular aspect (Fig. 4). After 3-months, atrophic scars were observed on the face and the trunk, erythematous atrophic scar with an anetodermal appearance and post-inflammatory hyperpigmentation without recurrence of lesions (Fig. 5).

## Discussion

Syphilis and SS association has been poorly reported. The first published case dated from 1986 when the authors analyzed whether syphilis was the cause of or simulated SS.^7^ Regarding syphilis, it is known as a great imitator given the wide variety of clinical presentations.^4^ An unusual form is nodular syphilis, which presents with erythematous plaques and nodules, whose histopathology exam frequently shows granulomas and plasma cells, compatible findings with one of our patient’s biopsies. The latter, added to characteristics skin lesions and treponemal tests, are consistent with the clinical picture of secondary nodular syphilis.^5^ In relation to SS, it is diagnosed based on clinical, analytical, and histological criteria. The criteria were proposed in 1986 and reviewed by Von den Driesch in 1994, where states that to make the diagnosis, two major criteria and two minor criteria must be met.^9^ In our case, the patient meets two major criteria (sudden appearance of painful erythematous nodules and plaques; dense neutrophilic infiltrate) and the 4 minor criteria (fever, association with infection, increased inflammatory parameters, and good response to systemic corticosteroids), confirming the diagnosis of SS.

The particularity of this clinical case is based on the rare association of these two pathologies, added to an unusual presentation of nodular syphilis and the presence of two characteristic histological patterns in the same patient. To our knowledge, there are no reports of cases that comply with both the clinical and histology of nodular syphilis to date, and also with a diagnostic criteria for SS. The clinical importance of this first report highlights the need, in the case of polymorphic clinical presentations, to perform multiple biopsies on the diverse lesions, which allows us to obtain more information regarding the studied pathology. In addition to carrying out the infectious etiological search for syphilis in patients with symptoms compatible with SS, since, despite its low clinical frequency, it implies a sexually transmitted disease with successful medical treatment.

## Financial support

None declared.

## Authors’ contributions

Claudio Escanilla: Design of the study and acquisition of data.

Yerco Goldman: Design of the study, acquisition of data and, drafting the article.

Francisco Bobadilla: Interpretation of data, drafting the article, and revising it critically.

Laura Segovia: Interpretation of data.

## Conflicts of interest

None declared.
